# Intervention Program to Improve Grief-Related Symptoms in Caregivers of Patients Diagnosed With Dementia

**DOI:** 10.3389/fpsyg.2021.628750

**Published:** 2021-02-11

**Authors:** Jorge Bravo-Benítez, Francisco Cruz-Quintana, Manuel Fernández-Alcántara, María Nieves Pérez-Marfil

**Affiliations:** ^1^Mind, Brain, and Behavior Resarch Center (CIMCYC, Centro de Investigación Mente, Cerebro y Comportamiento), University of Granada, Granada, Spain; ^2^Faculty of Psychology, University of Granada, Granada, Spain; ^3^Department of Health Psychology, University of Alicante, Alicante, Spain

**Keywords:** ambiguous grief, sorrow, dementia, Alzheimer's disease, caregiver, grief, intervention program

## Abstract

The objectives of the present study were to adapt a grief intervention program to family caregivers of patients with dementia, and assess its effectiveness in improving the symptoms of grief and other health-related variables. The intervention was based on Shear and Bloom's grief intervention program, with the necessary adaptations for use in the grieving process for a family member's illness. A total of 52 family caregivers of individuals with dementia participated. They were evaluated using a battery of self-report measures assessing grief, overload, resilience, post-traumatic growth, experiential avoidance, health-related quality of life, and benefits of care. The results suggest that the program is effective in improving grief symptoms, caregiver burden, resilience, post-traumatic growth, and quality of life of family caregivers. It is necessary to create and implement interventions targeting caregivers' feelings and manifestations of ambiguous grief, because there is a lack of programs providing an efficient solution for the mental and physical health of caregivers, and because of the human and socioeconomic cost involved in neglecting this group.

## Introduction

Dementia is a major cause of disability and dependency among elderly individuals worldwide. It can be overwhelming not only for the individuals who suffer from it, but also for their caregivers and family members (World Health Organization, 2019). The risk of developing dementia increases as individuals age, and the incidence of this illness doubles every 5 years after the age of 65 (Daviglus et al., 2010). Alzheimer's disease is one of the most common dementia and is characterized by significant deficits in memory and in different neuropsychological functions, has become a major health problem in the world.

Generally, patients with dementia will need specific care, with the caregiving role usually taken on by a family member (Bangerter et al., 2019). The figure of the caregiver is fundamental because of their involvement in the quality of life of the individual concerned and because they are the main source of information on the patient's state of health for health professionals (Fundación Sanitas, 2016). Caregiving tasks take up most of the caregiver's time, which may negatively affect their social, occupational, emotional, and family aspects of life (Piccini et al., 2012). This can cause them to neglect their own needs during the course of care, organizing everything according to the patient's demands. It has been found that family caregivers suffer from a greater number of physical and mental health problems compared to the general population (Kiely et al., 2008). High levels of anxiety and depression have been reported, as well as frequent use of psychotropic drugs, as a way of coping with the complex situation of caring for a patient with these characteristics (Piccini et al., 2012).

The gradual deterioration of the family member's state due to dementia is experienced as a gradual loss of the care recipients themselves. In other words, caregivers have to cope with a series of non-fatal, non-time-limited losses, an experience that is highly stressful and has been associated with a whole host of health problems (Rubin et al., 2019). In general, caregivers' grief remains unrecognized by their social environment and health institutions. Different models have tried to account for this specific type of loss, which has been referred to as “anticipated grief,” “ambiguous loss,” or “dementia grief.” Blandin and Pepin (2017) describe dementia grief loss as an anticipated grief produced in response to a series of cyclical losses of different magnitudes, characterized by a high degree of ambiguity and significant changes in the caregiver's identity. Boss (2016) also outlines the central role of ambiguity, identifying two types of ambiguous loss. The first type of ambiguous loss is when individuals perceive themselves to be physically absent, but psychologically present, and the second type is when individuals perceive themselves to be physically present, but psychologically absent, as is the case with dementia. This type of ambiguous loss, known as “saying goodbye without leaving,” can be considered as one of the most distressing and challenging aspects of the experience of caring (Boss, 2016) and distinguishes the experience of grief from someone else's dementia caregiver. Since the loss can be intangible or uncertain, the grieving process for family members can easily become complicated (Pauline and Boss, 2009).

In the literature, an increase in the intensity of caregiver stress preceding the physical death of the individual with dementia is reported. This stress can often be equal to or greater than the levels of grief stress after their death (Noyes et al., 2010). In addition, caregivers who experience higher levels of stress before the death of the patient are at greater risk of experiencing health complications after the death of the patient (Givens et al., 2011; Chan et al., 2013; Shuter et al., 2014). There is also evidence to suggest that the intensity of the grieving process shares risk factors with caregiving overload, such as the stage of illness and behavioral problems, but there also appear to be factors specific to the experience of loss (Liew et al., 2019). In this line, it could be pointed out that ambiguous loss is characterized by factors that inherently make the grieving process difficult (Boss, 2016), as this is a process that takes place gradually over time, without a fixed pattern and without being interpreted by the person as grief (Doka, 2008).

It has also been suggested that, unlike losses from death, grieving over non-fatal losses may not be socially recognized or openly supported, and may fall into the category of disenfranchised grief (Doka, 2008). Caregivers are deprived of the right to express their grief openly and may experience greater difficulties in adjusting to and overcoming the loss. Even though some studies have considered this phenomenon to be chronic grief (Sanders and Corley, 2003; Pauline and Boss, 2009; Noyes et al., 2010), the research suggests that little is known about the grieving process of caregivers of individuals with dementia (Doka, 2008; Chan et al., 2013; Arruda and Paun, 2017).

Despite this, recent models, such as the Two-Track Model of Dementia Grief (Rubin et al., 2019), have identified an entire series of variables that interact with each other and shape this grieving experience. The authors highlight four dimensions: (1) the characteristics of the individual diagnosed with dementia (diagnosis, severity, and symptom pattern); (2) the objective circumstances of the individual being cared for and of their caregiver (objective burden of care, losses associated with caregiving, ambiguity regarding the future); (3) the contextual factors relating to care (psychological resources and socio-demographic aspects); (4) the responses and coping mechanisms of the family system. This last dimension includes variables such as caregiver health levels, e.g., anxiety, depression, health-related quality of life, meaning of life, growth, positive changes, or resilience (Rubin et al., 2019).

As shown in a recent review, a very limited number of grief-focused interventions have been identified for caregivers of dementia patients (Arruda and Paun, 2017). The few interventions conducted pre-death focused on working on the emotional health of caregivers while their family members with dementia were still alive (Boerner et al., 2004; Schulz et al., 2006; Haley et al., 2008; Holland et al., 2009; Bergman et al., 2011) and were guided by stress models (Schulz et al., 2003; Mittelman et al., 2004). In addition, the utilization of cognitive-behavioral therapy targeting this type of loss is producing very promising results with respect to well-being, health, and symptoms of grief (Meichsner and Wilz, 2018; Meichsner et al., 2019a,b).

However, there are few interventions based on a caregiver grief model designed specifically to improve the emotional health of caregivers of dementia patients in manifestations of grief both before and after the death of the care recipient.

In the context of grief interventions, Shear's K. (2010) treatment of complicated grief has been shown to be effective and to have sustained effects over time in clinical trials (Papa et al., 2013; Rosner et al., 2014; Shear and Bloom, 2017). Complicated grief treatment is a16-session evidence-based psychotherapy developed to release and facilitates a bereaved person's natural adaptive response. This program is based on the cognitive-behavioral therapy model, and also includes interpersonal therapy techniques and motivational intervention. The program aims to intervene in the processes that are supposed to maintain a maladaptive grieving process, therefore it focuses on three basic objectives: properly process the experience and integrate the loss in the life history, identify and change the problematic beliefs and interpretations of the process, and replace anxious-depressive avoidance strategies with more adaptive ones (Boelen et al., 2006). Three strategies are worked on: sharing information, promote self-observation and self-regulation, and rebuild the connection. Therapeutic objectives include: (a) advancing in the planning of future goals and rewarding activities, (b) reviewing the history of the death, (c) identifying the vital changes that the loss has produced, and (d) fostering continuous bonds through living memories. A detailed description of each of the 16 sessions can be found in Shear and Bloom (2017). Nonetheless, no studies have been identified where this intervention is applied to grieving processes not linked to losses from death.

The objectives of this study were to adapt a grief intervention program to family caregivers of patients with dementia and assess its effectiveness in improving their symptoms of grief and other health-related variables. It was expected that caregivers who participated in this intervention program would exhibit significant improvements in their overall perceived health, quality of life, as well as a significant decrease in maladaptive manifestations associated with grief.

## Methods

### Design

This study used a repeated measures quasi-experimental randomized controlled design with allocation of participants to either the intervention group (IG) or to the control group (CG) (on a waiting list). A general linear model for a 2 × 2 repeated measures design was used to perform the analysis. The two levels for the between-groups factor concerned whether or not a participant had participated in the intervention program (IG and CG), while the two levels for the within-subjects factor corresponded to the two assessment times (pre-intervention and post-intervention).

### Participants

Fifty-two family caregivers of patients with dementia from the A.F.A. ALTAAMID Center (Association of Relatives of Patients with Alzheimer's disease) in the city of Granada, Spain, participated in the study. Of these 52 family members, 27 participated in the program (IG), and 25 did not receive any intervention (CG, on a waiting list). The participants were randomly allocated to one group or the other.

The inclusion criteria for study participation were: being the primary caregiver of a family member with any type of dementia; being aged 18 or above; consenting to participate in the program and being available to do so. The exclusion criteria for both groups were: experiencing difficulty with testing and participating in the program; currently receiving psychological and/or psychiatric treatment. The diagnosis of dementia in the family member had to have been made by a neurologist.

The mean age of the family caregivers was 63.88 years (SD = 17.55; range: 21–89), of which 21.15% were male and 78.85% were female. Thirty of the family caregivers (57.69%) were the spouse of the patient, 34.62% were their children, and 7.69% were other relatives. Most of them had a partner at that time and lived together (71.1%). In reference to their level of education, 7.69% no education, 26.92% had primary education, 19.23% had secondary education, and 46.15% had higher education. Regarding their employment status, 28.85% had a remunerated job, 50% were retired, 15.38% did household chores, and 3.85% were unemployed. With respect to the family's monthly income, 17.31% earned the minimum inter-professional wage in Spain (SMI in Spanish), 38.46% earned between 1 and 2 times the SMI, 25% earned between 2 and 3 times the SMI, and 19.23% earned more than 3 times the SMI.

Eight of the 52 family caregivers had previously been diagnosed with an affective or mood disorder. Sixty-three percent (63.46%) of the individuals with dementia were being cared for by only one caregiver, and 36.54% were being cared for by more than one caregiver. There were no significant differences in these variables between the CG and the IG (see Table 1).

**Table 1:** Sociodemographic characteristics of the sample.

**Variables**	**Intervention group (*n* = 27)**	**Control group (*n* = 25)**	**χ^**2**^*/t***
	**Mean (SD) or *n* (%)**	**Mean (SD) or *n* (%)**	***p***
Age	66.59 (17.25)	60.96 (17.56)	0.252
Gender			0.845
Males	6 (22.2%)	5 (20%)	
Females	21 (77.8%)	20 (80%)	
Relationship			0.139
Spouse	19 (70.4%)	11(44%)	
Son	7 (25.9%)	11 (44%)	
Others	1 (3.7%)	3 (12%)	
Co-existence			0.629
With a partner	20(74.1%)	17 (68%)	
Without a partner	7 (25.9%)	8 (32%)	
Level of studies			0.087
No studies	4 (14.8%)	0 (0%)	
Primary studies	9 (33.3%)	5 (20%)	
Secondary studies	5 (18.5%)	5 (20%)	
University studies	9 (33.3%)	15 (60%)	
Employment situation			0.447
Active	5 (18.5%)	10 (40%)	
Retirees	15 (55.6%)	11 (44%)	
Housework	5 (18.5%)	3 (12%)	
Unemployed	1 (3.7%)	1 (i4%)	
Monthly salary			0.288
IMW	4 (14.8%)	5 (20%)	
Between 1 and 2 IMW	11 (40.7%)	9 (36%)	
Between 2 and 3 IMW	9 (33.9%)	4 (16%)	
More than 10 IMW	3 (11.1%)	7 (28%)	
Number of caregivers			0.099
1	20 (74.1%)	13(52%)	
More than 1	7 (25.9%)	12 (48%)	

*IMW, Interprofessional minimum wage*.

### Instruments

The caregivers were assessed using the following instruments:

a) An interview to collect personal and socio-demographic data from the participants: their level of education, monthly family income, employment status, degree of kinship, the number of family members caring for the dependent relative, and whether they had ever been diagnosed with any psychiatric illness.
b) The Caregiver Grief Scale (CGS) (Meichsner et al., 2016), which measures the caregivers' manifestations of grief. Consisting of 11 items of Likert-type format with 5 categories, with a range that goes from 1 (totally disagree) to 5 (totally agree). The full scale and its subscales were shown to have high levels of internal consistency (Cronbach's α between 0.67 and 0.89) and high levels of construct validity. The scale includes four factors that reflect different aspects of caregiver grief: emotional pain (painful emotions related to the loss), relational loss (losses related to the relationship), absolute loss (anticipation of the future without the person), and acceptance of loss (acceptance of dementia and open expression of the grief). For the present study a back-translated version from English to Spanish was used, with reliability values ranging from α = 0.55 to α = 0.85 (emotional pain α = 0.62, relational loss α = 0.77, absolute loss α = 0.85, acceptance of loss α = 0.55) and an overall Cronbach's α of 0.85.
c) The Caregiver Burden Interview (CBI) (Zarit et al., 1980). The Spanish adaptation by Martín et al. (1996) was used. This scale assesses the stress and subjective overload perceived by the caregivers of dependent individuals. It consists of 22 Likert-type scale items with 5 frequency values ranging from 1 (never) to 5 (almost always). The internal consistency of the scale is α= 0.91 and its test-retest reliability is 0.96.
d) The Connor-Davidson Resilience Scale (CD-RISC) (Connor and Davidson, 2003). The Spanish adaptation by Crespo et al. (2014) was used. It consists of 10 Likert-type items of 5 categories that cover scores from 0 (absolutely) to 4 (almost always). This scale has a high level of internal consistency as measured with the Cronbach's α statistic (0.90). With respect to convergent and divergent validity, overall scores show positive correlations between CD-RISC and measures of self-esteem as well as with caregiver perception of self-efficacy. CD-RISC is shown to be inversely correlated with depression, anxiety, and caregiver burden.
e) The Acceptance and Action Questionnaire (AAQ-II) (Hayes et al., 2004). The Spanish adaptation by Mairal (2004) was used. This questionnaire assesses experiential avoidance and psychological flexibility. Consisting of 10 items of Likert type format with 7 categories that cover a score range from 1 (completely false) to 7 (completely true). It has a good level of internal consistency (as measured with Cronbach's α= 0.88), construct validity, discriminant validity, and external validity.
f) The Post-Traumatic Growth Inventory (PTGI) (Tedeschi and Calhoun, 1996). The Spanish adaptation by Castro et al. (2015) was used. It consists of 21 items that assess the perception of personal benefits in survivors of a traumatic event. It has a Likert-type response format with 6 categories, in a score range from 0 (no change) to 5 (very high degree of change) in a positive sense: the higher the score, the greater the perceived change. This instrument has a Cronbach's alpha of 0.95. Adaptations of the PTGI have found structures that vary from one to four factors, including the three-dimensional structure proposed by the original theoretical model. In Spain, there are studies corroborating the one-factor approach (Costa Requena and Gil Moncayo, 2007), as well as studies that have found a bifactor model consisting of three specific factors and one general factor (Rodríguez-Rey et al., 2016; Garrido-Hernansaiz et al., 2017).
g) Positive Aspects of Caregiving (PAC) (Tarlow et al., 2004). This measure assesses the benefits of providing care and has good internal consistency values. It consists of 11 items evaluated on a Likert scale from 1 (strongly disagree) to 5 (strongly agree). The original version showed adequate values of reliability (α = 0.89), as well as the Spanish adaptation, with Cronbach's alpha values of α = 0.82 (Las Hayas et al., 2014).
h) The SF-36 Health Survey (SF-36) (Ware and Sherbourne, 1992). The Spanish adaptation by Alonso et al. (1995) was used. This survey assesses perceived health: physical functioning, limitations due to physical problems, bodily pain, social functioning or role, mental health, limitations due to emotional functioning, vitality, energy or fatigue, and general health perception. It consists of 90 items that explore 9 psychopathological dimensions. Each item is valued according to a Likert-type scale with different scores in which the discomfort perceived in the last 7 days must be indicated. Internal consistency, as measured with the Cronbach's alpha statistic, ranges from 0.70 in the pain dimension to 0.90 in physical functioning. With respect to external validity, it has been shown to be significantly correlated with existing scales measuring similar constructs.

### Procedure

Firstly, the study was approved by the Ethics Committee on Human Research of the University of Granada, Spain (Ref.: 359/CEIH/2017). Subsequently, the research proposal was presented to the management team of the A.F.A. ALTAAMID Center. Once approved, the members of the center were contacted, were informed of the purpose of the research, and were asked for their collaboration. Fifty-two relatives of ~80 patients showed interest in taking part in this study. Two groups were formed, the IG and the CG, depending on whether participants were to follow the intervention program or not. The participants were randomly allocated to one group or the other. All the participants signed a written informed consent form and completed the assessment tests in a single session, always in the same order: the socio-demographic data interview; the SF-36 Health Survey; the Caregiver Grief Scale (CGS); the Caregiver Burden Interview (CBI); the Connor-Davidson Resilience Scale (CD-RISC); the Post-Traumatic Growth Inventory (PTGI); the Acceptance and Action Questionnaire (AAQ-II); and the Positive Aspects of Caregiving (PAC).

The assessments were conducted using the facilities at the ALTAAMID Center, with an approximate duration of 60 min each.

There were as many IGs as the sample size obtained, forming groups of 5 (three groups) and 6 (two groups) participants.

Subsequently, the caregivers in the IG received 10 one-and-a-half hour intervention sessions over the course of two and a half months, once per week. The intervention was implemented in the same facilities where the assessments were conducted. The intervention was based on the guidelines of Shear and Bloom's grief intervention program (2017). The intervention was adapted to fit the characteristics of the study population, i.e., it was adapted to fit the grieving process for a family member's illness. The changes that have been made to adapt the original version of the program are as follows: (a) the number of sessions, in the original format is 16, in the current program there are 10 sessions), (b) the duration of the sessions, 45–60 min become 90 min, (c) the type of grief that the program addresses does not focus on grief due to death, but grief due to illness of a loved one, (d) the theme of the imaginal exposure techniques that in this case, it focuses on different aversive moments in the interaction with the patient, and (e) the inclusion of a mutual aid group. Table 2 shows the content of each session of the intervention program.

**Table 2:** Contents of the ten sessions of the intervention program.

**Session number**	**Content**
1	- Group rules and presentation of the intervention - Participants' introductions - Psychoeducation: disenfranchised grief - Training in self-registration
2	- Description of the SUDS procedure (Subjective Units of Distress Scale) - Imaginal exposure - Working on rewarding activities - Working on goals
3	- Imaginal exposure - Working on personal resource management - Discussing avoided situations. Information
4	- Imaginal exposure - Discussing specific memories - Working on avoided situations - Working on rewarding activities and goals
5	- Imaginal exposure - Discussing specific memories
6	- Imaginal exposure - Working on avoided situations - Hot spots (remembering moments of exposure with high SUDS scores)
7	- Imaginal exposure - Discussing personal changes - Hot spots (remembering moments of exposure with high SUDS scores)
8	- Imaginal exposure - Discussing positive aspects in life
9	- Imaginal exposure - Role-playing and empty-chair technique with the affected family member - Anticipating and planning for painful dates/situations
10	- Imaginal exposure - Summary of the treatment - Identifying and dealing with feelings about the end of treatment. Encouraging acceptance of the new situation and developing the new bond - Discussing the potential for joy and satisfaction in life and positive feelings about working with patients - Goodbye

The following techniques were used: imaginal exposure and *in vivo* exposure, cognitive restructuring, behavioral rehearsals, and social skills training. The main objectives were to facilitate the acceptance of both the new situation and the consequences of the loss, to foster the bonds they had with their family member, and to promote strategies for participating in activities that would increase their levels of satisfaction and quality of life. Among the program's most characteristic strategies are the following: (a) imaginal exposure to different aspects of the situation, with special emphasis on exposure to those moments that caused them the highest levels of anxiety (hot spots); (b) managing personal resources and developing coping strategies to meet the demands of their family member in an efficient manner, while reducing the perceived gap between demands and available resources and reducing the perceived emotional impact of caregiving; (c) reinterpretation of situations that were previously avoided so that caregivers perceive themselves as more motivated and as having higher levels of self-efficacy with respect to coping; (d) promotion of rewarding activities based on the caregivers' interests and desires, and systematization of activities; (e) establishment of medium- and long-term goals and objectives, identifying obstacles and seeking alternative ways to achieve them; (f) promotion of social support through the establishment of a mutual support group among the participants, in which they were encouraged to remain after the intervention program.

At the end of each session, proposals for homework were made, and the following sessions began by reviewing these proposals.

Once the intervention finished, the IGs and the CG went through the two-session assessment protocol again, in the same order as described previously. The CG engaged in the daily activities that were being carried out in the association during the course of the intervention, which consisted of individualized counseling, informative talks related to the symptomatology of their family member, the promotion of their daily functioning, and the management of the behavioral problems which are characteristic of this illness. In a second phase, the participants of the CG who wished to do so, had the opportunity to complete the intervention program (CG meaning remaining on a waiting list).

Both the assessment and the implementation of the program were conducted by the same researcher, an expert in providing care to individuals with dementia.

### Statistic Analyses

Data were analyzed using IBM SPSS for Windows, version 22.0. Descriptive analyses were performed: for quantitative variables, means and standard deviations were used; for categorical variables, frequencies were calculated. Between-group differences were analyzed using the *t*-test for independent samples and the χ^2^ test. Linear models for repeated measures (Wilks' λ) were used to assess the effect of the program. In all cases, the assumptions of homogeneity of variances were taken into account (Levene's test). The effect size was calculated with Cohen's *d*. The statistical significance threshold was set at *p* < 0.05.

## Results

We present the results of the between-groups comparisonsbased on the assessment time for the different variables (see Tables 3, 4). In Tables 3, 4 we present the means, standard deviations, effect sizes (Cohen's *d*), and results obtained from between-groups differences, the assessment time, and the interactions between the variables. The dependent variables were caregiver grief (CGS), caregiver overload (CBI), resilience (CD-RISC), acceptance and action (AAQ-II), positive aspects of caregiving (PAC), post-traumatic growth (PTGI), and perceived health (SF-36).

**Table 3:** Differences between groups in the scales of grief, burden, resilience, experiential avoidance, post-traumatic growth, and positive aspects of caregiving.

**Variable**	**Group**	***Mean (SD)***	***Mean (SD)***	**Effect**	**Factor**	***F***	***p***
		**Pre**	**Post**	**Size**			
CGS emotional pain	Control	9.16 (3.26)	9.68 (3.29)	0.16	Time	1.95	0.169
	Intervention	11.22 (3.07)	9.52 (3.13)	0.55	Time × Group	6.89	0.011^*^
					Group	1.50	0.226
CGS relational loss	Control	11.00 (3.61)	10.48 (3.94)	0.14	Time	3.23	0.078
	Intervention	12.44 (2.91)	11.52 (2.99)	0.31	Time × Group	0.25	0.616
					Group	2.16	0.148
CGS Absoluteloss	Control	9.24 (3.74)	10.24 (3.81)	0.26	Time	0.04	0.849
	Intervention	12.44 (3.13)	11.26 (3.32)	0.37	Time × Group	5.08	0.029^*^
					Group	6.28	0.016^*^
CGS acceptance to loss	Control	6.04 (2.75)	6.16 (2.61)	0.04	Time	2.02	0.161
	Intervention	7.56 (1.93)	6.48 (2.34)	0.66	Time × Group	3.16	0.081
					Group	2.50	0.120
CBI	Control	47.80 (17.71)	51.96 (20.43)	0.22	Time	0.02	0.889
	Intervention	57.78 (18.22)	53.07 (15.96)	0.28	Time × Group	5.21	0.027^*^
					Group	1.43	0.237
CDRISC	Control	29.60 (9.17)	26.04 (9.99)	0.37	Time	0.10	0.755
	Intervention	23.74 (8.28)	27.89 (8.09)	0.51	Time × Group	16.96	0.000^**^
					Group	0.77	0.384
AAQII	Control	16.76 (10.79)	16.64 (9.90)	0.01	Time	4.37	0.042^*^
	Intervention	25.89 (11.95)	21.22 (10.23)	0.42	Time × Group	3.94	0.052
					Group	6.18	0.016^*^
PTGISF relationship with others	Control	5.72 (3.22)	4.88 (2.83)	0.28	Time	4.05	0.049^*^
	Intervention	6.33 (2.48)	5.37 (3.26)	0.33	Time × Group	0.02	0.891
					Group	0.64	0.428
PTGISF new possibilities	Control	4.72 (3.13)	4.20 (3.09)	0.17	Time	0.48	0.491
	Intervention	4.37 (3.00)	5.40 (3.37)	0.32	Time × Group	4.36	0.042^*^
					Group	0.29	0.590
PTGISF personal strength	Control	5.48 (3.25)	4.84 (3.00)	0.20	Time	0.38	0.540
	Intervention	5.33 (3.09)	6.41 (3.25)	0.34	Time × Group	5.93	0.019^*^
					Group	0.79	0.379
PTGISF spiritual change	Control	3.60 (3.43)	3.36 (3.49)	0.07	Time	0.12	0.724
	Intervention	3.67 (3.54)	4.19 (3.64)	0.14	Time × Group	0.93	0.338
					Group	0.25	0.621
PTGISF appreciation of life	Control	5.44 (3.43)	5.96 (3.30)	0.15	Time	2.89	0.096
	Intervention	6.30 (2.66)	7.04 (2.30)	0.30	Time × Group	0.09	0.767
					Group	1.77	0.189
PTGISF total	Control	24.96 (11.07)	22.76 (10.75)	0.20	Time	0.01	0.960
	Intervention	26.00 (10.58)	28.33 (11.67)	0.21	Time × Group	2.93	0.093
					Group	1.43	0.237
PAC	Control	41.04 (10.99)	40.40 10.34)	0.06	Time	4.94	0.031^*^
	Intervention	43.48 (6.81)	48.26 (6.85)	0.70	Time × Group	8.46	0.005^**^
					Group	5.09	0.028^*^

*CGS, Caregiver Grief Scale; CBI, Zarit Burden Interview; CD-RISC, The Connor-Davidson Resilience Scale; PTGI, Post-traumatic Growth Inventory; AAQ-II, Acceptance and Action Questionnaire II; and PAC, Positive Aspects of Caregiving. r = effect size*.

* p < 0.05;

** *p < 0.01*.

**Table 4:** Differences between groups in the SF-36 Scale.

**Variable**	**Group**	***Mean (SD)***	***Mean (SD)***	**Effect**	**Factor**	***F***	***p***
		**Pre**	**Post**	**Size**			
SF36 general health	Control	14.88 (3.15)	15.76 (2.52)	0.31	Time	2.01	0.163
	Intervention	15.89 (3.75)	16.15 (3.01)	0.08	Time × Group	0.60	0.444
					Group	0.81	0.372
SF36 physical health	Control	27.32 (3.52)	26.48 (4.27)	0.22	Time	1.16	0.286
	Intervention	22.59 (5.81)	24.56 (4.96)	0.37	Time × Group	7.24	0.010^*^
					Group	7.56	0.008^**^
SF36 physical role	Control	7.36 (1.22)	6.80 (1.68)	0.39	Time	0.01	0.951
	Intervention	6.00 (1.90)	6.59 (1.78)	0.32	Time × Group	4.72	0.035^*^
					Group	4.22	0.045^*^
SF36 emotional role	Control	5.56 (1.61)	4.80 (1.50)	0.49	Time	0.45	0.507
	Intervention	4.74 (1.40)	5.15 (1.46)	0.29	Time × Group	4.88	0.032^*^
					Group	0.54	0.464
SF36 social function	Control	6.04 (1.24)	5.88 (0.78)	0.16	Time	0.92	0.342
	Intervention	6.33 (1.57)	6.00 (1.36)	0.23	Time × Group	0.11	0.737
					Group	0.71	0.403
SF36 body pain	Control	5.00 (2.31)	5.36 (2.96)	0.14	Time	4.52	0.039^*^
	Intervention	6.33 (3.45)	4.74 (2.54)	0.53	Time × Group	11.34	0.001^**^
					Group	0.23	0.630
SF36 vitality	Control	14.76 (2.37)	14.80 (1.53)	0.02	Time	0.94	0.338
	Intervention	14.30 (2.46)	14.89 (2.22)	0.25	Time × Group	0.71	0.402
					Group	0.13	0.715
SF36 mental health	Control	19.36 (2.53)	18.84 (2.66)	0.20	Time	1.02	0.318
	Intervention	17.11 (2.74)	18.44 (2.53)	0.50	Time × Group	5.28	0.026^*^
					Group	4.79	0.033^*^
SF36 physical component	Control	54.56 (3.71)	54.40 (4.28)	0.04	Time	0.12	0.731
	Intervention	51.48 (5.04)	52.04 (4.28)	0.12	Time × Group	0.39	0.534
					Group	6.48	0.014^*^
SF36 emotional component	Control	45.48 (4.65)	44.56 (4.16)	0.21	Time	0.21	0.650
	Intervention	42.74 (3.61)	44.22 (4.04)	0.39	Time × Group	3.80	0.057
					Group	2.55	0.048^*^
SF36 total	Control	102.00 (6.14)	100.64 (5.84)	0.23	Time	0.53	0.470
	Intervention	96.44 (5.82)	99.79 (6.08)	0.56	Time × Group	5.68	0.021^*^
					Group	6.09	0.017^*^

*SF-36 = Health Scale*.

* p < 0.05;

** *p < 0.01*.

**Figure 1:**
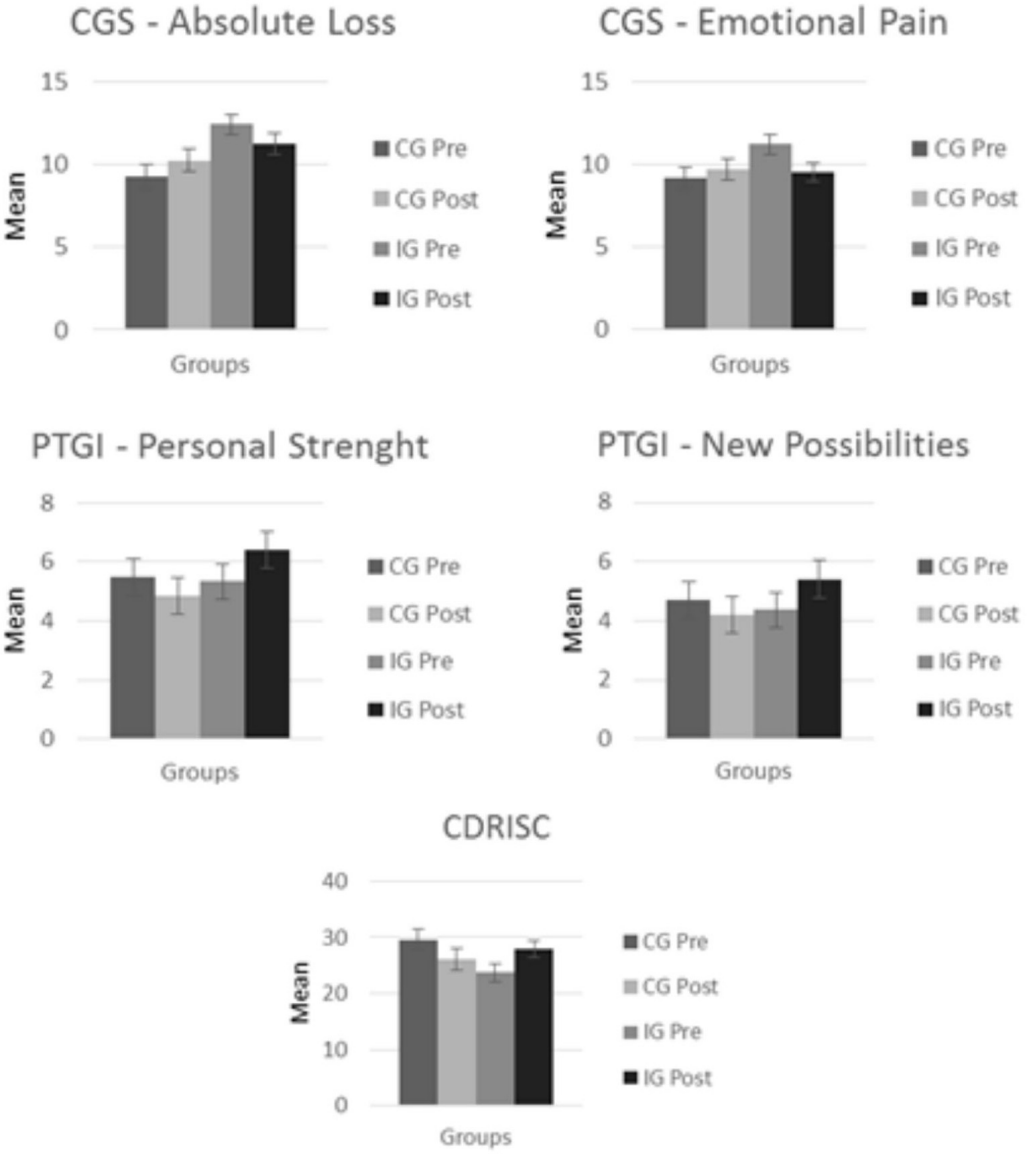
Mean values in each group for the grief, post-traumatic growth, and resilience variables.

**Figure 2:**
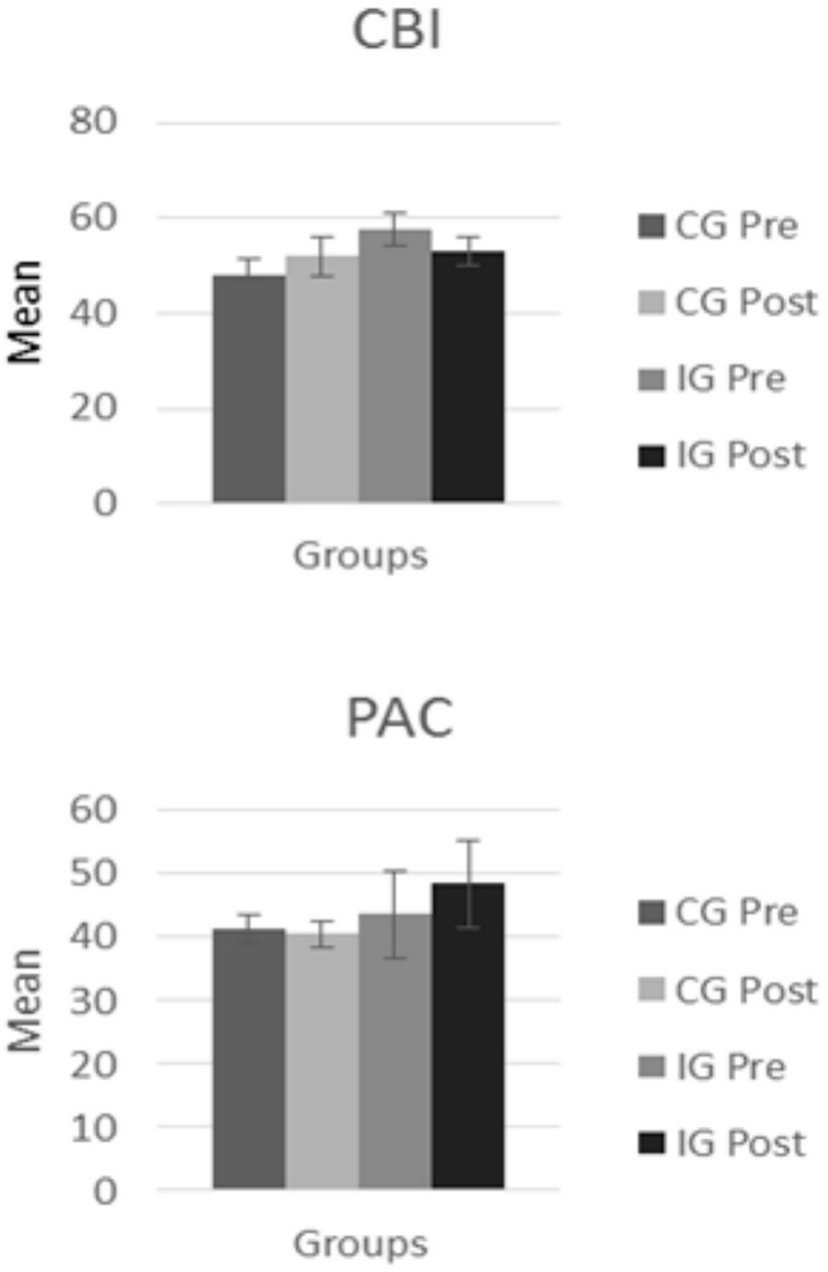
Mean values in each group for the caregiver burden and the positive aspects of caregiving.

Significant Time × Group interactions were observed between six of the variables assessed: manifestations of grief, caregiver overload, resilience, some dimensions of post-traumatic growth, positive aspects of caregiving, and health-related quality of life (see Figures 1, 2).

With respect to the scales related to caregiver grief, we found that there were statistically significant differences in the scales CGS-Emotional pain [*F*_(1, 50)_ = 6.889; *p* = 0.011] and CGS-Absolute loss [*F*_(1, 50)_ = 5.080; *p* = 0.029] for the Time × Group interaction. In Emotional Pain variable, these differences indicate a decrease in the manifestations of grief-related emotions in the IG and an increase in emotional pain in the CG. In the Absolute Loss variable these differences indicated a decrease in the feelings of loss of meaning and desperation regarding the future loss in the IG, and an increase of them in the CG.

With respect to caregiver overload, we found statistically significant differences for the CBI in the Time × Group interaction [*F*_(1, 50)_ = 5.210; *p* = 0.027], indicating that there was an increase in caregiver overload between the first and second assessments in the CG, while in the IG, caregiver overload decreased after the intervention.

In the case of resilience (CD-RISC), statistically significant differences were found in the Time × Group interaction [*F*_(1, 50)_ = 16.961; *p* < 0.001]. These differences show that, while a decrease in resilience was observed in the CG between the first and second assessments, this value increased in the IG.

When observing the scores obtained on the experiential avoidance (AAQ-II), the statistically significant differences within the factors of Time (*F*_(1, 50)_ = 4.373, *p* = 0.042) and Group (*F*_(1, 50)_ = 6.178, *p* = 0.016) could be highlighted. These results indicate that, after the intervention, there is a decrease in these values in the IG, while the score remains relatively unchanged in the CG.

The PTGI-SF results show differences in the scales New possibilities [*F*_(1, 50)_ = 4.356; *p* = 0.042] and Personal strength [*F*_(1, 50)_ = 5.929; *p* = 0.019] for the Time × Group interaction, indicating a decrease between the first and second measurements for the CG and an increased between these measurements for the IG.

We also found significant differences between measurements of positive aspects of caregiving taken using the PAC in the Time × Group interaction [*F*_(1, 50)_ = 8.465; *p* = 0.028]. These results indicate a pattern of change between the two different assessments for each of the two groups, with a decrease being observed in the CG and an increase being observed in the IG.

As for the SF36 scales, we found statistically significant differences in Physical Health, Physical Role, Mental Health, and the Total Scale in the Time × Group interaction (see Table 4). In all cases, these differences are explained by the fact that, in the second assessment, the CG showed a decrease in perceived health, and the IG showed an increase. Significant differences in the Time × Group interaction also appear for the Emotional Role scale [*F*_(1, 50)_ = 4.885; *p* = 0.032], indicating a decrease in Emotional Role scores between the two assessments within the CG, and an increase in Emotional Role scores between the two assessments within the IG. Finally, the effect sizes for the IG are generally moderate, except for those of the PAC scale (*d* = 0.70), which are moderately high.

## Discussion

The objectives of this study were to adapt a grief intervention program to family caregivers of patients with dementia and assess its effectiveness in improving their symptoms of grief and the other health related variables. The results suggest that the program is effective in improving grief symptoms, caregiver burden, resilience, post-traumatic growth, and quality of life of family caregivers.

Most of previous interventions for family caregivers have been proven to be effective in improving the quality of life and emotional health of caregivers, they have not usually taken into account the symptoms related to grief during the process of deterioration of their loved ones. In the literature, it is reported that the lack of attention paid to the symptoms of this type of grief increases the probability that the caregiver develops health problems after the death of their family member (Givens et al., 2011; Chan et al., 2013; Shuter et al., 2014) and the grieving process may become complicated (Pauline and Boss, 2009).

The current program has been proven to be an effective tool for improving the well-being and quality of life of family caregivers. These benefits also have a positive effect on the care of their affected family members. In particular, the results obtained show that, after participating in the program, caregivers exhibited significant reductions in symptoms associated with grief, and also in their levels of caregiving-related stress (emotional pain associated with grief, feelings of absolute loss, subjective overload in the performance of their caregiving role, and experiential avoidance).

One of the most frequently reported outcomes in intervention studies with caregivers of dementia patients concerns the effects of these interventions on caregiver burden (Wasilewski et al., 2017; Wilz et al., 2018). These studies point out that any intervention with a planned end may not be enough to ease the burden on caregivers, as their situation becomes more complex and difficult over time (Chiu et al., 2009). In the present study, the program is not only shown to be effective in reducing the subjective burden in the performance of the caregiving role, but it also promotes a number of factors that facilitate coping with caregiving tasks (resilience, perception of the caregiver's role, and ability to adapt to adverse situations). Promoting these factors has a positive impact on the quality of care delivered to individuals with dementia and may have a protective effect on the caregivers' management of their caregiving process throughout the course of the illness. In the present study, participants perceive that they have further resources available (knowledge and support) to be able to face the ups and downs of their transition to a better physical and mental state, and to be able to provide care and improve their willingness and behaviors with respect to the duties of caring for their family member, thus providing them with better quality care (more affectionate care, more thorough care, etc.). These changes, in turn, result in a decrease in the behavioral problems usually displayed by the person being cared for.In other studies, the perception of improved physical health has been associated with a decrease in demand for healthcare and a reduction in use of psychotropic drugs, prescribed or otherwise, in caregivers (Kiely et al., 2008).

As we have pointed out, our program significantly reduces the symptoms associated with grief. One of the symptoms that is modified is experiential avoidance. It is reported in the literature that avoidance is one of the symptoms associated with barriers to processing grief (Blandin and Pepin, 2017) and has significant long-term negative consequences on caregivers (Meichsner et al., 2019a). Shear M. K. (2010) had already pointed out that working on avoidance is not routinely considered in clinical situations. Our results show that Shear's program, the basis of our study, yields favorable results with respect to this symptom in the study population, i.e., caregivers of individuals with dementia, in line with recent intervention studies (Meichsner et al., 2019b). The dimensions of emotional pain and the absolute of the loss also showed an improvement in the IG. These aspects are related to the painful grief-related emotions and to the anticipation of the future without the loved one. However, the aspects of relational loss and the acceptance of the loss were not statistically significant between groups. Future studies using the present intervention program should also include tasks and exercises focused on the relationship (including communication and daily activities) and to the acceptance and open expression of grief (Meichsner et al., 2016). Another topic which is present in the literature on interventions for caregivers of individuals with dementia is their mode of implementation (i.e., in groups or individually) and the modules and techniques used in different interventions/programs. Moderate to strong effects have been reported, with mixed results regarding the longevity of its effectiveness, with respect to individual interventions regarding grief prior to the patient's death (Ott et al., 2010; Paun et al., 2015). Recent studies show that interventions for grief management may be conducted with positive results by other means, such as via e-mail (Chiu et al., 2009), via telephone (MacCourt et al., 2016; Wilz et al., 2018), or via the Internet (Meichsner and Wilz, 2018). Although the effects are not conclusive, one of the benefits of this type of intervention is that these environments provide caregivers with flexible access to support, without the problems they often encounter in individual or group settings, or being unable to attend as they cannot leave the care recipient alone. One drawback of this way of conducting the intervention is that the caregiver needs to have access to the internet and a certain level of digital literacy (Meichsner et al., 2019b). In group interventions, it becomes evident that, among other things, the use of education modules, the identification of changes in situations of grief and loss, and also of coping mechanisms are useful to the group (Sanders and Sharp, 2004). Our intervention program is conducted in groups, and involves the use of the following techniques: imaginal exposure, *in vivo* exposure, cognitive restructuring, behavioral rehearsals, and social skills training. The results show that group work promotes social support and group expression, and helps in the reduction of the level of discomfort experienced by the caregivers by promoting identification with others, which contributes to processing grief.

This program covers care aspects of care that are very disabling for the individuals who suffer from them (emotional lability, grief from repeated losses, drug abuse, social isolation, personal dissatisfaction, hopelessness about the future, feelings of worthlessness, etc.), and which have not been targeted by any specific intervention before. The following benefits of the program can be highlighted: (a) the program attends to the grieving processes of family caregivers who, until then, had not had these attended to; (b) levels of adherence to the program are high, and this program is applicable to any caregiver, regardless of the stage of the illness; (c) it does not require many material or professional resources for implementation; (d)it promotes proactive changes in coping mechanisms with the role of the caregiver and the symptoms of grief; (e) it promotes changes that have an impact at the societal, family, and personal levels; (f) it incentivizes and facilitates the engagement in actionsthat already existed prior to the need for self-realization; (g) it creates long-lasting bonds of support between caregivers participating in the program. It is necessary to increase the resources allocated to researching and developing programs for caregivers in which the focus is on the the symptoms of grief experienced during the care process, because, today, regardless of the direct costs inherent to supporting individuals with dementia (Rojas et al., 2010), the socioeconomic and human costs of neglecting this group are on the rise (Galende et al., 2021). It is important to provide comprehensive care tailored to the specific needs of caregivers, including reducing caregiver overload, improving their well-being and quality of life, and providing interventions for their manifestations of grief.

Among the main limitations of this study, we could highlight the heterogeneity of the sample in terms of the characteristics of the dementia patients being cared for. In particular, there were differences in their stage of illness, level of cognitive impairment, level of dependence, and the severity of their behavioral problems. Future studies are needed to analyze how the characteristics of the patients may influence the effectiveness of the intervention. It would also be desirable to be able to incorporate into the study design the different degrees of kinship the caregivers may have with their care recipients. Another limitation refers to the fact that it has not been possible to assess the time of care, which can influence the physical and emotional effects on caregivers. Moreover, participants need to be monitored to ensure that the caregivers are able to maintain, after completing the program, the positive changes that they have made.

In conclusion, the grief intervention program used in this study, which is based on the guidelines of Shear and Bloom (2017), has been shown to be effective for use in family caregivers of patients with dementia. The program has resulted in an improvement in caregivers' overall perceived health, quality of life, and well-being, as well as a significant decrease in frequency of maladaptive manifestations of grief.

## Data Availability Statement

The raw data supporting the conclusions of this article will be made available by the authors, without undue reservation.

## Ethics Statement

The studies involving human participants were reviewed and approved by 359/CEIH/2017. The patients/participants provided their written informed consent to participate in this study.

## Author Contributions

JB-B, MP-M, and FC-Q: conceptualization, methodology, software, validation, formal analysis, investigation, resources, data curation, visualization, and project administration. JB-B, MP-M, MF-A, and FC-Q: writing original draft preparation, writing review and editing, and funding acquisition. MP–M and FC-Q: supervision. All authors contributed to the article and approved the submitted version.

## Conflict of Interest

The authors declare that the research was conducted in the absence of any commercial or financial relationships that could be construed as a potential conflict of interest.
